# No one knows what attention is

**DOI:** 10.3758/s13414-019-01846-w

**Published:** 2019-09-05

**Authors:** Bernhard Hommel, Craig S. Chapman, Paul Cisek, Heather F. Neyedli, Joo-Hyun Song, Timothy N. Welsh

**Affiliations:** 1 Institute of Psychology, Cognitive Psychology Unit and Leiden Institute for Brain and Cognition, Leiden University, Leiden, the Netherlands; 2 Faculty of Kinesiology, Sport, and Recreation, University of Alberta, Edmonton, Alberta Canada; 3 Department of Neuroscience, University of Montreal, Montreal, Quebec Canada; 4 School of Health and Human Performance, Dalhousie University, Halifax, Nova Scotia Canada; 5 Department of Cognitive, Linguistic and Psychological Sciences, Brown University, Providence, RI USA; 6 Centre for Motor Control, Faculty of Kinesiology and Physical Education, University of Toronto, 55 Harbord Street, Toronto, ON M5S 2W6 Canada

**Keywords:** Attention, Motor control, Selection, Sensorimotor, Decision making, Phylogenetic, Intention, Evolution, Parietal cortex, Superior colliculus

## Abstract

In this article, we challenge the usefulness of “attention” as a unitary construct and/or neural system. We point out that the concept has too many meanings to justify a single term, and that “attention” is used to refer to both the *explanandum* (the set of phenomena in need of explanation) and the *explanans* (the set of processes doing the explaining). To illustrate these points, we focus our discussion on visual selective attention. It is argued that selectivity in processing has emerged through evolution as a design feature of a complex multi-channel sensorimotor system, which generates selective phenomena of “attention” as one of many by-products. Instead of the traditional analytic approach to attention, we suggest a synthetic approach that starts with well-understood mechanisms that do not need to be dedicated to attention, and yet account for the selectivity phenomena under investigation. We conclude that what would serve scientific progress best would be to drop the term “attention” as a label for a specific functional or neural system and instead focus on behaviorally relevant selection processes and the many systems that implement them.

## Introduction

“Everyone knows what attention is” (James, 1890) is one of the most popular quotes from William James and certainly the most famous statement about human attention.^1^ We argue, however, that the overuse and popularity of this statement in cognitive research has been detrimental to progress – that in fact, no one knows what attention is. More specifically, we argue that the concept of “attention” is one of the most misleading and misused terms in the cognitive sciences. In the present paper, we stake the position that the term “attention” should be abandoned and the nature of the research in this area be re-conceptualized to focus on the subsets of processes and mechanisms that lead to task-specific performance. Similar positions have been proposed and discussed previously (see Anderson, 2011; Di Lollo, 2018; Hommel & Colzato, 2015; Krauzlis, Bollimunta, Arcizet, & Wang, 2014; Mole, 2011). The present paper reaffirms and expands this position by placing particular and new emphasis on the interconnected and integrative nature of the human sensorimotor information processing systems. This emphasis on integrated sensori-cognitive-motor processes takes inspiration from the synthetic approach to understanding “cognition” (Hommel & Colzato, 2015) and a proposed phylogenetic refinement of the scientific approach to understanding behavior (Cisek, 2019 [this issue]).

In the present paper, we start by discussing and outlining the central problem with the way “attention” has been conceptualized and studied thus far. We make the case for adopting a synthetic approach to studying cognitive phenomena wherein the focus is on the subset of processes and mechanisms that have been attributed to and investigated under the umbrella of “attention,” rather than on “attention” as one overarching concept. To bolster our analysis of the state of affairs, we present two test cases. In the first, we examine the debate about the conceptual distinction between attention and intention, and show that this debate fails to adequately account for the available data. In the second, we review research on selection and reward history to show how conventional analytic approaches to solving this problem are ineffective. As an alternative to the analytic approach, we provide a brief review of the phylogenetic evolution of the human brain (for an expanded account, see Cisek, 2019 [this issue]) and show how selective attention emerged as just one necessary consequence of the challenges facing animals behaving in the natural world. In the end, we conclude that the traditional analytic attempt to lump many diverse empirical observations under one common umbrella called “attention” and to try to explain all of them by referring to one coherent attentional system has actually failed, and should be replaced by a more synthetic approach. This synthetic approach focuses on, and starts with, ecologically relevant mechanisms and processes and then tries to account for as many phenomena (“attentional” or not) as possible.

## The concept of attention

We are not the first to raise concerns about problems with the term “attention.” Multiple authors have highlighted the tendency to reify attention, creating circular explanations for empirical results (Anderson, 2011; Di Lollo, 2018). Another common criticism is that multiple processes underlie what is typically labeled as “attention” (Di Lollo, 2018; Hommel & Colzato, 2015). Mole (2011) highlights that James’ statement came at the time where there was debate among theorists as to whether the main role of attention was in thinking, perceiving, or acting. James’ contemporary, F.H. Bradley, produced one of the earliest criticisms of the concept of attention, titling his essay “Is There Any Special Activity of Attention.” In brief, his position was that there were too many examples of phenomena labeled “attention”, with little concern about the processes underlying such phenomena (Bradley, 1886). Now, over 130 years later, there is a continued and heightened need to question the role of “attention”, the use of the term “attention,” and a search for what “attention” is (e.g., Busemeyer, Gluth, Rieskamp, & Turner, 2019; Gottlieb, 2012). We reaffirm these positions and further suggest that compartmentalizing “attention” and then searching for the “attentional system” hinders the development of a comprehensive understanding of human behavior because it ignores integrated, parallel, and reciprocal relationships among sensory, cognitive, and action processes.

Before we explain our main position and arguments, we would like to emphasize that the theoretical problems that we highlight are particularly visible with respect to the concept of attention, but by no means restricted to that concept (Hommel, 2019a). For example, very similar arguments to those that we present in the following have been put forward to question the concept of memory. Decades of research on human memory have seen an ever-increasing number of memory systems that were thought to represent separable aspects of memory performance, which then were thought to be explained by the existence of corresponding memory systems, with rather limited contributions to a mechanistic understanding of the underlying processes (Bechtel, 2008). As recent considerations suggest, however, the various types of memory may not at all reflect the operations of separable dedicated systems, but rather stand for different byproducts of normally functioning cognitive systems (Buckner & Schacter, 2004), and emerged at different times during the evolution of our species (Murray, Wise, & Graham, 2017). Similar arguments have been put forward for the concept of emotion (Barrett, 2017; Hommel, 2019b) and may be developed for other concepts as well, including “cognition” itself (Cisek, 2019). We focus here on attention because we believe that at least some of the related phenomena are best understood in terms of the kinds of interactions between sensory, motor, and cognitive phenomena that are the focus of this special issue.

Theorizing about human attention suffers from at least three main problems. First, the concept of attention invites misconceptions of one coherent set of cognitive or neural operations, depending on one’s level of analysis, that all contribute to what we call “attention” (e.g., Kahneman, 1973). Second, the concept of “attention” can also easily be misunderstood as *both* an important explanandum that psychology is rightly expected to explain *and* the explanans that is supposed to form the explanation – thus rendering the latter a pseudo-explanation. And, third, the concept is thought to distinguish a particular set of cognitive or neural operations from other, seemingly different sets of operations, such as those related to decisions, intentions, motivations, emotions, and, of particular relevance to the present special issue, action planning and execution. As we show in the expanded discussion of these three points in the following paragraphs, all these assumptions are incorrect.

First, let us start by considering which phenomena researchers have, historically speaking, been trying to explain when using the term “attention.” According to some traditional and conventional views, “attention” is the set of cognitive/neural mechanisms responsible for maximizing the efficient utilization of our limited capacities to process, store, and retrieve information. However, consultancy of introductory textbooks (e.g., Eysenck & Keane, 2000) and the internet reveals a dramatic variety of abilities attributed to attention: the ability to select external events for further internal processing (focused attention); ignore misleading information and/or an irrelevant location (selective attention); process irrelevant information (involuntary attention); selectively integrate information belonging to one event within and across sensory modalities (feature integration); prioritize processing of events from a particular location (spatial attention); systematically search for a target event (visual search); perform multiple tasks at the same time (divided attention); control the spatial parameters of eye movements (selective attention for action); prioritize one goal over others (goal-centered attention); prioritize one object, memory item, or conscious representation over others (object-centered attention); and consolidate information for later use and concentrate in anticipation of a possible event over some time (sustained attention). At face value, it seems highly unlikely that the same set of functional/neural mechanisms are involved in, and responsible for, this broad variety of phenomena (Allport, 1993), and a bulk of behavioral and neural evidence confirms that most subfunctions can be dissociated from each other (e.g., Fan, McCandliss, Sommer, Raz, & Posner, 2002). Accordingly, it is unsurprising that no theory has been suggested so far that comes even close to providing a coherent account of all phenomena sailing under the label of “attention.”

Second, the term “attention” is often used to capture both the problem and the solution of cognitive processing; i.e., to describe both the phenomenon one aims to explain and the mechanism proposed to provide the explanation. For instance, the term attention is used to refer to the consequences of both “voluntary” and “involuntary” factors in favoring the representation of one event having a stronger impact on decision making and action than representations of other events (e.g., Yantis, 1998). But the concept of attention is also used to refer to the system, mechanism, or ability to deal with (or avoid) the consequences of such unequal potencies of representations to drive behavior (e.g., Broadbent, 1958). Along the same lines, attention is considered by some to represent the critical capacity limitation, the cognitive bottleneck that needs to be accounted for and explained (e.g., Pashler & Johnston, 1998), while still others consider attention to be the cognitive means to deal with such bottlenecks (e.g., Bundesen, 1990). These conceptual confusions have created a situation in which it is no longer clear what the to-be-explained problem actually is (do we have a cognitive bottleneck that we need to make the best of, or do we have too much information we need to choose from?), and whether attention is a concept that refers to the problem or to the solution. This runs into the danger that research and theorizing on attention is based on circular reasoning (attentional phenomena are explained by assuming and pointing to attentional systems) rather than on a deeper mechanistic understanding of how the observed phenomena are causally produced (Krauzlis et al., 2014).

Third, research on attention has followed, and suffered from, the common analytical approach to psychological functioning (see Hommel & Colzato, 2015, for a more detailed discussion of this issue). The analytic approach comprises a search for an exhaustive definition (which, given the diversity of the subfunctions of attention, is impossible), the identification of assumed subfunctions (e.g., overt vs. covert, early vs. late, focused vs. divided, voluntary vs. automatic attention, etc.) with separable functional and neural processes, and the concentration of research on tasks and subfunctions rather than actual processes. The problem of this analytic approach is that it underestimates and overlooks commonalities between subfunctions and, in a wider perspective, commonalities with other concepts. For instance, the very fact that we use concepts like attention, decision making, intention, emotion, and motivation in different situations and theoretical contexts by no means implies that the underlying functional and neural *processes* are different and separable. Indeed, attempts to systematically distinguish the processes “underlying” attention from the processes that do not, often fail to produce any coherent consensus. In the following section, we outline one exemplar case of the failure of the analytic approach – the attempt to separate “attention” from “intention.”

## Attention versus intention: A failed dichotomy

Most researchers agree that the posterior parietal cortex represents the core of the neural substrate of selective attention, and is a key node of an “attentional network” (Corbetta & Shulman, 2011; Posner & Dehaene, 1994; Ptak, 2012). In particular, individual neurons in the posterior parietal cortex appear to reflect the locus of attention (Bisley & Goldberg, 2010; Robinson, Goldberg, & Stanton, 1978) and parietal damage often leads to phenomena of spatial neglect (Bartolomeo, 2007; Corbetta & Shulman, 2011). However, a separate line of research implicates these same regions of the brain in processes related to movement control (Mountcastle, Lynch, Georgopoulos, Sakata, & Acuna, 1975; Snyder, Batista, & Andersen, 1997). In particular, regions of the posterior parietal cortex are strongly and reciprocally interconnected with parts of the frontal lobe that are involved in the planning and guidance of movement (Johnson, Ferraina, Bianchi, & Caminiti, 1996; Markov et al., 2014), individual neurons are strongly modulated by the type of action performed with respect to identical stimuli (Cui & Andersen, 2007; Snyder et al., 1997), and inactivation of the posterior parietal cortex causes biases in free-choice tasks (Christopoulos, Kagan, & Andersen, 2018), but not decisions based on visual evidence (Katz, Yates, Pillow, & Huk, 2016).

These apparently contradictory findings have fueled a heated and persistent debate, now in its fifth decade, on whether the posterior parietal cortex is involved in guiding “attention” or whether it reflects the individual’s “intention.” As is typical for the dominant analytical approach to psychological science, researchers have tried to resolve this debate by defining the concepts of attention and intention in ways that make them appear mutually exclusive: “Attention” is what restricts the inflow of sensory information to cognition, what enters conscious thought for further processing, whereas “intention” is the output of cognition, the will (free or otherwise) to perform a specific action. Defined in this way, the two appear like distinct concepts that must be dissociable through careful experimental design. And yet, after decades of work by some of the world’s most accomplished neuroscientists, a clear dissociation of the function of posterior parietal cortex remains elusive. A prominent review expressed this frustration many years ago, suggesting that “current hypotheses concerning parietal function may not be the actual dimensions along which the parietal lobes are functionally organized; on this view, what we are lacking is a conceptual advance that leads us to test better hypotheses” (Culham & Kanwisher, 2001).

To escape this rather uncomfortable state of affairs, some researchers have argued for a more integrative view on attention and intention. A particularly promising approach is the pre-motor theory, which argues that shifts of attention are triggered by sub-threshold saccadic commands in oculomotor areas and, conversely, shifts of attention in space lead to action planning (e.g., Rizzolatti, Riggio, Dascola, & Umilta, 1987). Support for the former idea has come from a large number of observations including: (a) behavioral studies showing that attention and eye movements are strongly linked behaviorally (e.g., Deubel & Schneider, 1996; Kowler, Anderson, Dosher, & Blaser, 1995; Sheliga, Riggio, & Rizzolatti, 1995); (b) fMRI studies of visual attention showing activation in eye-movement areas for attention tasks (e.g., Beauchamp, Petit, Ellmore, Ingeholm, & Haxby, 2001; Corbetta et al., 1998; Nobre, Sebestyen, & Miniussi, 2000) and for movement activation (decoding) in retinotopically defined visual cortex during movement tasks (Gallivan, Chapman, Gale, Flanagan, & Culham, 2019); (c) stimulation studies showing that activation of neurons in the superior colliculus (SC), frontal eye field (FEF), and lateral intraparietal area (LIP) can change the focus of attention (Cavanaugh & Wurtz, 2004; Cutrell & Marrocco, 2002; Moore & Fallah, 2001; Muller, Philiastides, & Newsome, 2005); and (d) neurological studies of patients with attentional disorders following damage to the frontal cortex, parietal cortex, or midbrain (e.g., Husain & Kennard, 1996; Posner, Cohen, & Rafal, 1982; Posner, Rafal, Choate, & Vaughan, 1985; Sapir, Soroker, Berger, & Henik, 1999). Of particular relevance to the present purpose are behavioral studies revealing that perceptual discrimination at the goal location of an upcoming saccade is improved (Deubel & Schneider, 1996; Gersch, Kowler, & Dosher, 2004; Hoffman & Subramaniam, 1995; Peterson, Kramer, & Irwin, 2004). These studies demonstrate there is preferential processing of stimuli at the goal of a saccade just before the onset of the eye movement (presumably because “attention” has been shifted to the goal location).

Other research has extended the study of these action-attention interactions to manual actions, showing that planning and performing reaching and grasping movements prioritizes the processing of the target objects of these movements (e.g., Pratt & Abrams, 1994; Rizzolati, Riggio, & Sheliga, 1994; Tipper, Lortie, & Baylis, 1992; see also Wu, 2014). Even when the eyes remain fixated, perceptual discrimination is better at the to-be-reached goal than non-goal locations (Baldauf & Deubel, 2008; Baldauf, Wolf, & Deubel, 2006; Deubel & Schneider, 2003; Deubel, Schneider, & Paprotta, 1998; Khan, Song, & McPeek, 2011). The “attentional impact” or prioritized processing associated with intended future movements goes beyond mere spatial prioritization because other studies have shown that moving or planning to move also facilitates the detection of action-related features of the object targeted by the movement. For example, preparing for a grasping movement facilitates the detection of size oddballs, while preparing for a pointing movement facilitates the detection of location oddballs (Fagioli, Hommel, & Schubotz, 2007; see also Craighero, Fadiga, Rizzolatti, & Umiltà, 1999). Other studies (Bekkering & Neggers, 2002; Moher, Anderson, & Song, 2015; Tipper, Meegan, & Howard, 2002; Weir et al., 2003; Welsh & Pratt, 2008; Welsh & Zbinden, 2009; see also Gallivan, Barton, Chapman, Wolpert, & Flanagan, 2015; Glazebrook, Welsh, & Tremblay, 2016; Yoxon, Constable, & Welsh, 2019 [this issue]) show that the processing of specific object features can be prioritized depending on the *relative* (i.e., task/action-specific) salience of those features for the to-be-performed action. That is, the same feature (e.g., orientation) can be prioritized in one action context (e.g., grasping), but not another action context (e.g., pointing). Hence, it is neither physical stimulus properties nor action goals alone that generate selectivity, but rather selectivity is shaped by the reciprocal and iterative interactions between these factors. These findings thus suggest that multiple functional and neural systems are involved in selective attention.

In addition to the interactive nature of stimulus properties and action goals in determining selection and prioritization of locations and features, it does not seem that selection stops solely within any putative attentional system. Indeed, neural activity related to multiple simultaneously active intentions to act at potential target locations, as well as the selection of the final target, has been identified in various structures more commonly associated with the planning and execution of actions, such as the dorsal premotor area, the parietal reach region, and the motor cortex (Cisek & Kalaska, 2005; Klaes, Westendorff, Chakrabarti, & Gail, 2011; Pesaran, Nelson, & Andersen, 2008; Scherberger & Andersen, 2007; Song & McPeek, 2010; Thura & Cisek, 2014). Behaviorally, the presence of multiple co-existing response representations and the dynamic selection of target from non-target stimuli and actions is also expressed through the spatiotemporal characteristics of reaching and grasping movements. Specifically, instead of the efficient straight and direct movements that one might anticipate if attentional selection had been completed prior to the intention to act, the trajectories of hand and eye movements veer towards or away from non-target stimuli depending on the timing and salience of the non-target stimuli (e.g., Chapman et al., 2010; Gallivan & Chapman, 2014; Howard & Tipper, 1997; Moher et al., 2015; Neyedli & Welsh, 2012; Song & Nakayama, 2006, 2008; Welsh, 2011; Welsh & Elliott, 2004; Wispinski, Gallivan, & Chapman, in press). Thus, the *characteristics of the physically executed action* actually reflect the “attentional” state of the target and non-target stimuli. Collectively, these data indicate that attention, selection, and intention are not readily separated in a set of discrete serial processes, but are more dynamic and continuous in nature and embedded within a densely interconnected, parallel processing system.

While more work needs to be done to synthesize these neural and behavioral observations into a coherent framework, it seems clear (to us) that the conceptual distinction between attention and intention is not sufficient to account for the variety of findings discussed here. The distinction fails to provide a meaningful contribution or framework for sorting the available findings into useful categories to stimulate further theorizing, and it also clashes with the demonstration of so many interactions between input processing and output generation. But what is the solution to this and the many other conceptual problems we are encountering in thinking about human attention (e.g., controlled vs. automatic processing; facilitation vs. inhibition, etc.)?

## A failed analytic solution: Selection and reward history

As noted above, the dominant analytical approach to psychological functioning begins with an exhaustive search for a definition of a concept, including the borders of where it differs from other concepts. So, to understand “attention,” one would tend to first define how it differs from “intention,” “decision-making,” “motivation,” etc. In view of a failure of this approach, as is obvious for the case of attention versus intention, two reactions are to be expected. First, one might consider the previous attempts to define attention and intention as flawed and try to improve the definitions by further reducing the conceptual overlap between the two concepts. For instance, one may further reduce the concept of attention to mere input selection and the concept of intention to output selection. Given that this would make it no longer apparent that such a reduced version of “attention” has anything to do with other “attentional” functions like integration, orientation, or vigilance, this would eventually call for dropping the concept – and the same argument holds for “intention.” On the positive side, this would prevent researchers from trying to find commonalities in processes and substrates that are unlikely to be found. On the negative side, however, there is no theoretical justification to pick just these functional aspects but not others. What looks like a definitional issue thus becomes a theoretical bias that is lacking justification.

Alternatively, one might search for hybrid approaches that allow for additional components and factors. A typical approach of this sort was the resource theory of attention, which triggered heated debates in the 1970s and 1980s (Kahneman, 1973; Navon, 1984). While the first approaches were simple and elegant by assuming one kind of resource that needs to be distributed over all mental work, the attempt to integrate an increasing number of unpredicted findings led to the invention of increasing numbers and types of separate resources. In the end, this made systematic predictions impossible (Navon, 1984), which is the main reason why this approach no longer plays an important role – except in the field of ego-depletion, where history seems to repeat itself (Friese, Loschelder, Gieseler, Frankenbach, & Inzlicht, in press). The main reason why hybrid approaches that simply lump together different factors are not overly successful rests in the fact that the respective factors are not truly integrated into a coherent framework.

A similar tendency can be seen with respect to selective attention, where Awh, Belopolsky, and Theeuwes (2012) have tried to integrate findings that are no longer consistent with the historical distinction between endogenous attention, which represents the prioritized processing of stimuli to which the agent “wants” to attend, and exogenous attention, which represents the prioritized processing of stimuli that are unrelated to the present action and goals. The history of distinguishing between endogenous and exogenous attention is very similar to the distinction between attention and intention. Each started out by trying to improve definitions about what the concept referred to, only to be faced later with the inability to systematically sort the available findings into two distinct categories. In a nutshell, endogenous attention is sometimes too automatic and exogenous attention is sometimes too dependent on the current prioritized stimulus feature or action goal to make this dichotomy fruitful and tenable (Awh et al., 2012; Folk, Remington, & Johnston, 1992; Hommel & Wiers, 2017). Awh et al. suggest solving this problem by adding a third variable – selection history – to the list of factors. In particular, the idea is that goals (the factor responsible for endogenous attention), salience (the factor responsible for exogenous attention), and selection history (a factor that does not seem to fit the previous dichotomy and is associated with previous selections and rewards generated by the selections) all contribute to selectivity by sending their output to an integrative priority map. Although this approach may account for many of the available findings, we are not convinced that it really solves the problem, but rather provides a patch that holds concepts together and, in the end, prevents or misdirects the search for suitable solutions. Instead, we suggest that a complete dismantling of the concept of attention is required.

At first there does not seem to be anything wrong with the idea that structures, or a singular structure, in the human central nervous system are devoted to collecting and integrating information that affects prioritized processing. One candidate structure is the superior colliculus, which is thought to reflect a priority map of stimuli in the visual field (Fecteau & Munoz, 2006). Importantly, and as required from the view that selectivity for behavioral relevance is the purview of the entire moving body, the superior colliculus is involved not just in eye movements, but in orienting movements of the eye, head, body, and hand (Gandhi & Katnani, 2011; Stuphorn, Hoffmann, & Miller, 1999). Emphasizing this point, Song and colleagues (Song, Rafal, & McPeek, 2011; Song & McPeek, 2015) found that the superior colliculus plays a causal role in target selection during manual reaching tasks, supporting the idea that the superior colliculus is part of a general-purpose target selection/orientation system (Nummela & Krauzlis, 2010; Song et al., 2011). On the other hand, there is no need to assume that the superior colliculus is the only map that integrates relevant information to steer attention, nor is it necessary to assume that all available information is integrated into that one map. As we argue below, the human brain can be considered to have many sources of selectivity and, in the end, it is the brain as a whole that does the integration. Given that this integration is the explanandum (the to-be-explained phenomenon), postulating the existence of one map that has no other function than achieving this integration seems to be one more attempt to “explain” a psychological phenomenon by positing the existence of a dedicated system whose only purpose is to somehow create that phenomenon.

Apart from this more general meta-theoretical problem, adding one more factor to a model that just assumes that integration takes place without explaining how that can be done is unlikely to guide further research. In the case of Awh et al. (2012), one reason is that selection history overlaps considerably with goal-induced endogenous selectivity and salience-induced exogenous selectivity. For instance, the fact that planning and carrying out particular kinds of actions systematically facilitates the processing of particular object features (e.g., of size and orientation for grasping, location for reaching: Bekkering & Neggers, 2002; Craighero et al., 1999; Fagioli et al., 2007) is unlikely to be genetically determined, but rather the consequence of learning and experience of selecting different features for grasping over the lifespan (Hommel, 2010). Indeed, prioritizing shape and orientation when grasping objects makes more sense than prioritizing color because those features are more likely to determine a successful or an unsuccessful grasp. Hence, establishing a bias for shape and orientation over color when grasping would be a functional adaptation. However, this influence implies that selection history affects how goals impact (endogenous) attention. Along the same lines, the relative salience of the visual dimension changes substantially during the first years of life (e.g., Suchman & Trabasso, 1966), which at least opens the possibility that selection history impacts salience.

While these arguments are fully consistent with Awh et al.’s suggestion to consider selection history as a third factor involved in attentional control, they also imply that the resulting three factors are not independent but strongly overlapping and intertwined – both empirically and conceptually. As we have tried to explain, these conceptual-overlap problems are unlikely to be resolved by more definitions. Rather, what is needed is a theory that not only assumes that integration takes place but that explains how that integration works.

Another reason why just adding selection history as an additional factor raises more questions than answers is that the concept itself is unclear, particularly in its overlap with other related factors beyond exogenous and endogenous control. One such factor that is intertwined with selection history is reward history. It is uncontroversial that previously rewarded stimuli receive preferential processing (Anderson, Laurent, & Yantis, 2011; Anderson & Yantis, 2012), suggesting that reward history is important in determining salience. Conventionally, stimuli must have been selected in order for the organism to have received a reward, thus conflating the two concepts. Awh et al. (2012) appear to acknowledge this tension, considering both previous reward and previous stimulus selections to be exemplars of “selection history” while facing the fact that selection history and reward history cannot be identical (given that previous selections might not have received reward). However, the fit of reward history into this joint category is much less obvious than this theoretical integration suggests since reward history is itself likely composed of many differentiable factors.

For instance, the preference for rewarding stimuli is stronger when the larger expected gain of the stimulus is due to an increased probability of receiving a reward than when there is a lower cost of failure (Neyedli & Welsh, 2015b). This finding suggests that what participants take to be rewarding is itself multifaceted – not just the magnitude of the reward, but also how likely it is that they will receive the reward. Furthermore, across a series of reach-decision experiments, a multitude of biasing factors have been observed including: reward value and probability (Chapman, Gallivan, & Enns, 2015a), the best option in a decision set (Wispinski, Truong, Handy, & Chapman, 2017), current level of accumulated wealth (Neyedli & Welsh, 2015a), the number of targets and not the perception of them (Milne et al., 2013), and how the number of choice-options is represented (Chapman et al., 2014). The problem here is similar to the one Awh et al. (2012) tried to solve: the definition of the concept under investigation is too restricted. In their case, endogenous and exogenous control were insufficient to account for the variety of pheomenena being ascribed to the concept of attention, so they added selection history. Selection history is itself decomposable into (at least) selection and reward history, and reward history is itself decomposable even further. Thus, the nature and the influence of reward is itself dependent on numerous contextual factors and the expression of the confluence of these factors is not easily captured in a unitary construct.

The picture gets even more complicated when one considers studies comparing the impact of rewards of equal magnitude but in opposite directions (e.g., positive/gain vs. negative/loss). For example, loss aversion, made famous by the work of Kahneman and Tversky (1979), shows that people treat potential losses as being more aversive than equivalent gains are rewarding. Interestingly, when decisions between positively and negatively rewarding stimuli are made rapidly (Chapman et al., 2015b), asymmetries in choice behavior are also observed, but go *opposite* to loss aversion. That is, in these situations, participants appear to be disproportionately drawn toward options giving gains, while the aversive impact of loss-related choices is attenuated. These findings highlight an additional complication – that different biases are likely to operate on different timelines. For instance, in the study of the asymmetry in decisions to go for a good option versus avoid a bad one, participants were biased toward positively valanced targets 100 ms earlier than they were biased to move away from negative ones (Chapman et al., 2015b). This finding echoes related work showing that more time is required to select optimally between visuomotor choices when they differ based on negative value information compared to when they differ based on the probability of reward (Neyedli & Welsh, 2015b).

Taken together, these and other findings strongly suggest that selection history is tightly interconnected with reward history and that neither of these concepts are particularly well understood. Hence, adding selection history to the two other not well understood concepts of exogenous and endogenous attention is unlikely to help much in understanding the mechanisms underlying human selective attention. To be clear, we are not advocating an alternative theory at this point, but rather we suggest an alternative theoretical perspective: Let us replace the analytical approach, which seeks to explain complex phenomena by first carefully defining them and then subdividing them into simpler elements, with a synthetic approach that considers how simple *mechanisms and functional processes*, each of which is itself behaviorally relevant, can together give rise to complex phenomena.

## A synthetic approach

A synthetic approach is valuable only insofar as it synthesizes elements that actually correspond to real biological processes at both neural and functional levels, and it is a significant challenge to figure out what those processes are. One powerful strategy for doing so – for keeping our synthesis close to biological reality – is to use evolution as a guide. This guidance can be done through a procedure of “phylogenetic refinement,” whereby one progressively elaborates a theory about how neural and behavioral processes evolved along a given lineage, always respecting constraints about the neural modifications and behavioral adaptations that appeared at each stage (see Cisek, 2019 [this issue]). For this reason, here we step away from the concept of “attention” and take a brief detour into the history of how the relevant neural circuits evolved in the lineage that leads to homo sapiens (humans). While it is often very difficult to know *why* a given modification took place in evolution, establishing *what* was the sequence of modifications can be constrained by a wealth of comparative and developmental data, leading to strong and testable hypotheses about how neural circuits and behavioral abilities evolved together.

**Fig. 1:**
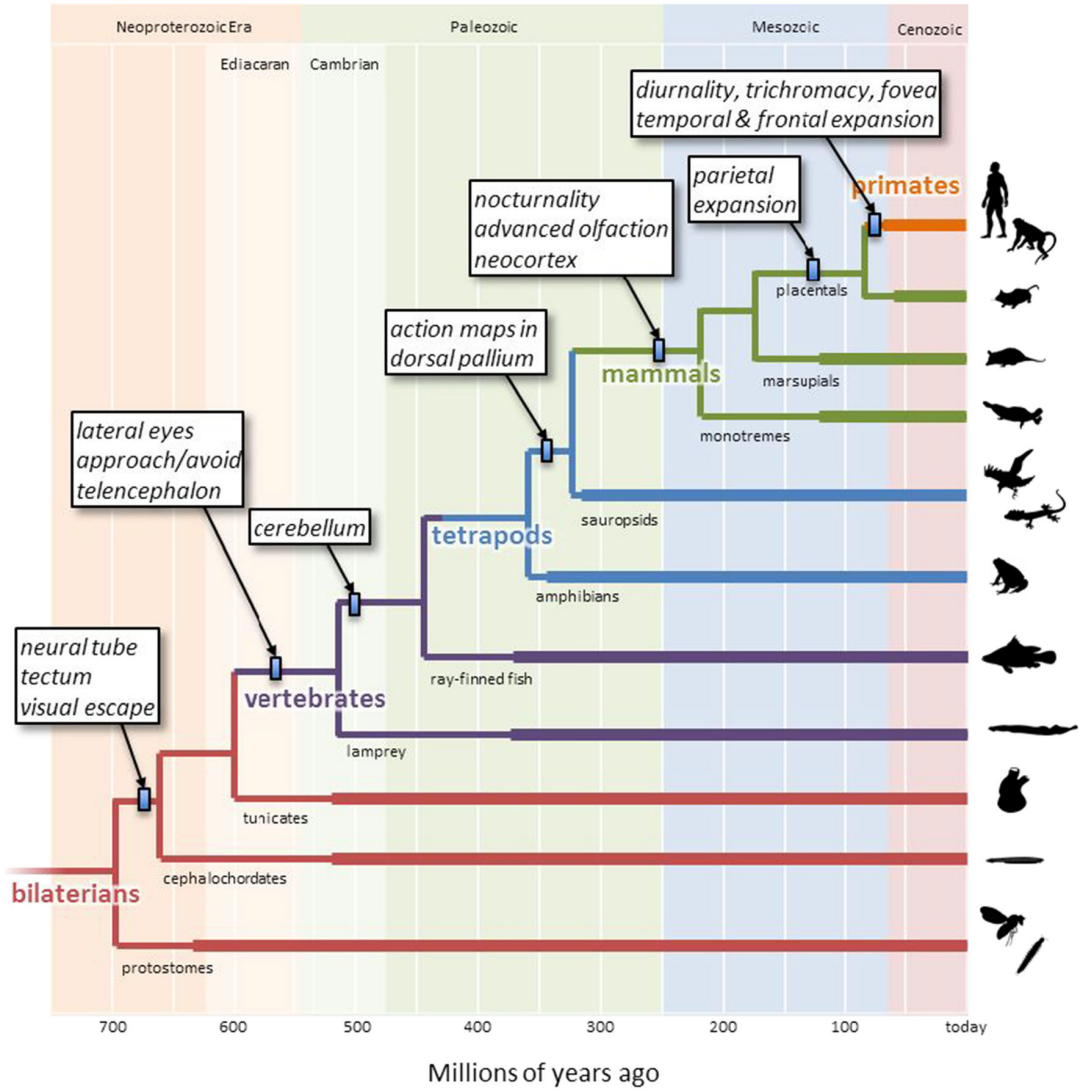
A reduced phylogenetic tree of bilaterally symmetric animals, exclusively emphasizing the lineage that leads to humans. Branch points represent some of the divergences between different lineages, with timing estimated on the basis of molecular clock analyses (Erwin et al., 2011). Thick lines indicate the presence of relevant fossil data (paleobiodb.org). Small rectangles indicate the estimated latest timing of innovations described in the boxes. Note that many branch points and lineages are omitted for clarity. Silhouettes along the right are from phylopic.org

**Fig. 2:**
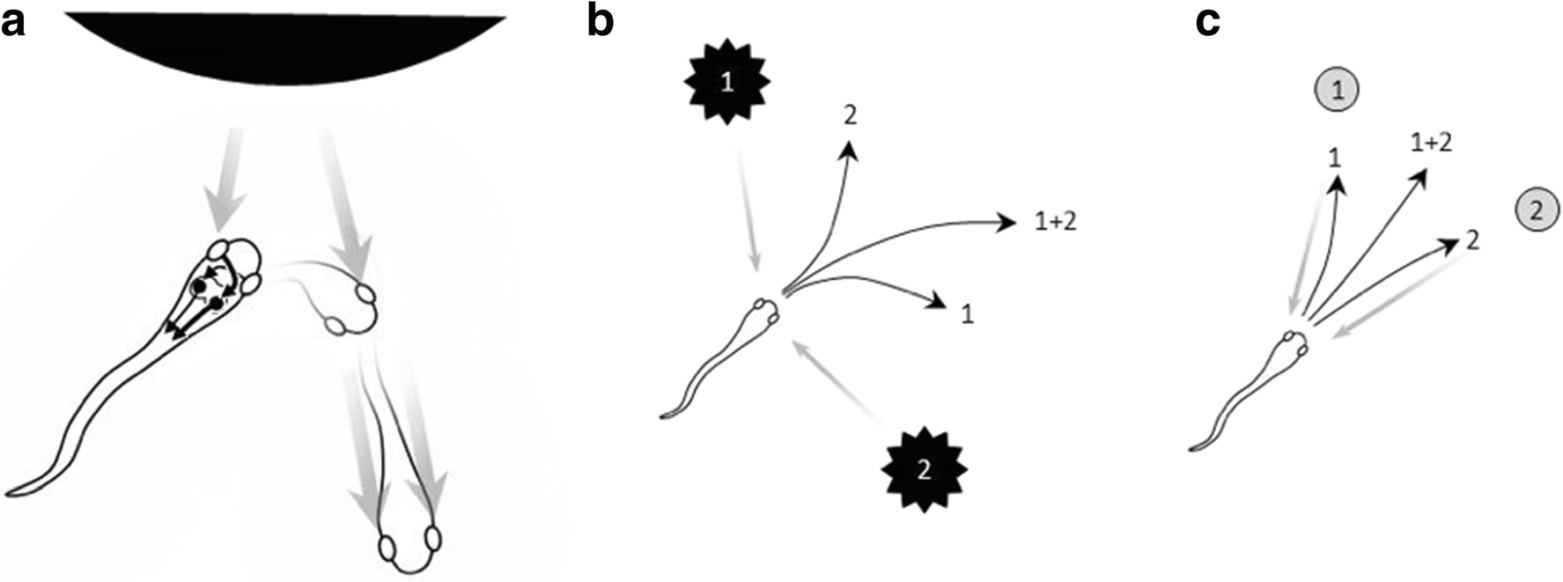
Circuits for avoidance and approach in a hypothetical early vertebrate. (**a**) In the avoidance circuit, visual information from the lateral eyes arrives in the contralateral tectum, which projects ipsilaterally to the midbrain locomotor regions. Thus, if a stimulus falls on the left eye, the locomotion will tend to turn to the right until stimulation is balanced and the body is oriented away from the stimulus. (**b**) Spatial averaging of escape directions (numbered arrows) away from two threatening stimuli (black stars) is an effective response. (**c**) For approach actions, spatial averaging is maladaptive, making winner-take-all dynamics necessary. B and C reused with permission from Cisek (2019)

The evolutionary history of spatial interaction along the primate lineage is a long and complex tale (Fig. 1). A major advance occurred during the Cambrian epoch, over 500 million years ago (Mya), with the elaboration of visually guided orientation behaviors. Our simple chordate ancestors possessed a visual escape circuit that involved projections from a single photosensitive patch in the rostral neural tube to a midbrain structure called the tectum, which projected to the spinal cord to generate locomotion (Lacalli, 1996, 2018). In the lineage leading to vertebrates, the photosensitive patch split into two lateral eye patches on both sides of the head (Butler, 2000). Because these eye patches projected *contra*laterally to the tectum, which projected *ipsi*laterally to the spinal cord, the circuit caused our ancestors to turn away from salient visual stimuli such as the shadow from an approaching predator (Fig. 2a). As the eye patches expanded, they folded into cups and formed a lens (Lamb, 2013), resulting in a two-dimensional retina that provided a topographic mapping of external stimuli. The tectum expanded in parallel, with a matched topographic map of space in its superficial layers and gradients of downstream projections in its deep layers. The result was an “action map” of oriented escape responses to threatening stimuli at specific locations in the external world.

Microstimulation studies reveal the presence of an organized map of oriented escape responses in the tectum of lamprey (Saitoh, Menard, & Grillner, 2007), a jawless fish whose ancestors diverged from ours about 550 Mya. These studies also reveal the presence of another action map, which lies within the rostral region of the tectum. This map is sensitive to space in front of the animal, and projects mostly *contra*laterally to the spinal cord, thereby producing orientation and approach actions (Jones, Grillner, & Robertson, 2009; Kardamakis, Saitoh, & Grillner, 2015). It is this latter tectal sub-circuit that is most relevant to attention and selection. In the avoidance circuit, multiple stimuli can engage multiple escape actions that can simply be averaged downstream to produce adaptive avoidance behavior (Fig. 2b). In contrast, averaging cannot work in an approach circuit, because the average response to two stimuli will cause the animal to miss both of them (Fig. 2c). Consequently, the approach circuit must *select* between actions, such that one completely suppresses the other. This kind of selection could be accomplished through lateral inhibition that produces “winner-take-all” dynamics (Grossberg, 1973; Mysore & Knudsen, 2011; Wang, 2002).

How is this related to attention? A few sentences after that famous phrase we quoted above, James wrote that attention “implies a withdrawal from some things in order to deal effectively with others.” That withdrawal from some stimuli to interact with another stimulus is indeed accomplished, quite literally, within the approach circuit of the rostral tectum. And while these simple circuits for governing interactive behavior may seem far removed from the higher cognition of humans, they are indeed the precursors to the mechanisms that control what has been called “selective attention.” The tectum is homologous to the human superior colliculus, which, as discussed earlier, is strongly implicated in both orienting gaze through eye and head movements and in controlling covert attention when gaze is stationary (Basso & May, 2017). Though much has developed in the central nervous system and the world since our lineage diverged from lamprey in the early Cambrian, both the approach and avoidance circuits of the tectum are still present in fish (Herrero, Rodriguez, Salas, & Torres, 1998) and in mammals (Comoli et al., 2012).

Eventually, our ancestors left the seas and some of them, the amniotes, adapted to a fully terrestrial lifestyle. This adaptation was accompanied by an expansion and lamination of the telencephalic pallium, an integrative olfactory, visual, and somatosensory region that would eventually give rise to the cerebral cortex (Aboitiz & Montiel, 2015; Striedter, 2005). In all mammals, the neocortex consists of two sheets (Finlay & Uchiyama, 2015), a dorsomedial sector that is spatially topographic and a ventrolateral sector that is non-topographic. In primates, the former includes a medial and dorsolateral prefrontal cortex, cingulate regions, all of the premotor, motor, sensorimotor, and parietal cortex, as well as the retrosplenial cortex. The latter includes parts of the lateral prefrontal cortex, orbitofrontal cortex, and all of the limbic cortex and the temporal lobe. Most relevant to the issue of attention is the dorsomedial sector of the neocortex, which is organized into a set of fronto-parietal circuits dedicated to different classes of species-typical actions (Graziano, 2016; Kaas & Stepniewska, 2016). In early mammals (300 Mya), this system was probably quite limited and consisted simply of medial circuits concerned with locomotion and lateral circuits concerned with head and mouth movements (Kaas, 2017). Each of these circuits processed sensory information in an idiosyncratic manner specialized for its specific type of action (e.g., space near the legs for locomotion, space near the snout for ingestion) and each projected to a specific set of relevant effectors. In a sense, each circuit was an “action map” analogous to the much older tectal systems for approach and avoidance, but guiding the much wider repertoire of task-specific interactions available in the mammalian niche.

**Fig. 3:**
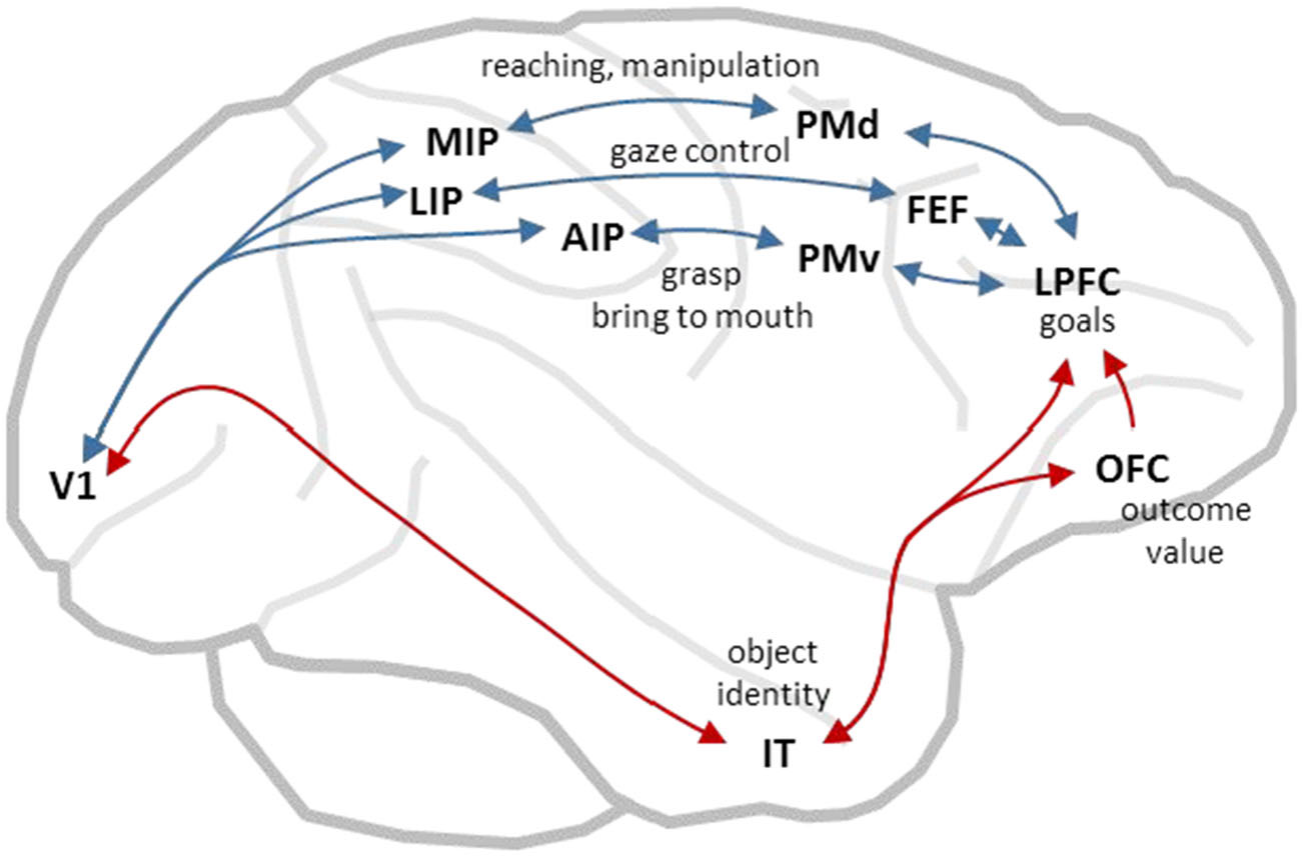
The primate cerebral cortex contains a set of parallel sensorimotor streams in the dorsomedial regions (blue arrows), each involved in a specific type of action using specific representations of space. All of these use information on object identity and outcome value, computed in the ventrolateral regions (red arrows), to select the actions most relevant given the current behavioral context. *AIP* anterior intraparietal area, *FEF* frontal eye fields, *IT* inferotemporal cortex, *LIP* lateral intraparietal area, *LPFC* lateral prefrontal cortex, *MIP* medial intraparietal area, *OFC* orbitofrontal cortex, *PMd* dorsal premotor cortex, *PMv* ventral premotor cortex, *V1* primary visual cortex

As the behavioral repertoire of mammals continued to expand, so did the dorsomedial neocortex, and there was a differentiation and specialization of action-specific maps of sensory space. In primates, expansion of the parietal cortex was particularly dramatic, yielding a variety of idiosyncratic representations of space particular to the needs of different action types (Andersen, Snyder, Bradley, & Xing, 1997; Stein, 1992) (see Fig. 3). For example, visually guided reaching actions involve the medial intraparietal cortex (Cui & Andersen, 2007; Kalaska & Crammond, 1995), which represents targets within reach with respect to the direction of gaze and the position of the hand (Buneo, Jarvis, Batista, & Andersen, 2002; Gallivan, Cavina-Pratesi, & Culham, 2009), and is interconnected with frontal regions controlling reaching, such as the dorsal premotor cortex (Johnson et al., 1996; Wise, Boussaoud, Johnson, & Caminiti, 1997). Grasp control involves the anterior intraparietal area (Baumann, Fluet, & Scherberger, 2009), which is sensitive to object shape and is interconnected with grasp-related frontal regions such as the ventral premotor cortex (Nakamura et al., 2001; Rizzolatti & Luppino, 2001). The control of gaze involves the lateral intraparietal area (Snyder, Batista, & Andersen, 2000), which represents space in a retinotopic frame (Colby & Duhamel, 1996; Snyder, Grieve, Brotchie, & Andersen, 1998), and is interconnected with frontal regions controlling gaze, such as the frontal eye fields and the superior colliculus (Paré & Wurtz, 2001) – taking advantage of the tectal orientation/approach system that has been steering animals since the Cambrian epoch.

In many situations, different fronto-parietal action streams will compete against each other. For example, one must make an all-or-none decision as to whether to burrow at the roots of one tree or instead walk over to another tree. In other situations, however, different fronto-parietal streams will be coordinated. For example, when a head/snout orientation system points at a target, that target is then made available to other behaviors, such as burrowing or biting. This availability becomes particularly important in primates, which evolved from tree-climbing insect eaters and developed large eyes with a central, high-resolution fovea, and acquired a taste for fruit. In such animals, the system for controlling the orientation of gaze takes on an executive role for many other visually guided behaviors. Selecting a target for gaze becomes part of selecting what to reach for or which branch to grasp to climb. It comes to serve much of the role traditionally ascribed to “selective attention.” Indeed, it has long been proposed that selective attention, both overt and covert, is closely related to the gaze orientation system and involves the same neural structures (Corbetta et al., 1998; Rizzolatti et al., 1987), including the posterior parietal cortex, the frontal eye fields, and the superior colliculus.

And so, this brief foray into the long journey of primate evolution has brought us back to selective attention and to the posterior parietal cortex, but with a different perspective on both. The question is not whether the posterior parietal cortex plays a role in something called “attention” or something else called “intention,” but how the posterior parietal cortex fits within a broader system that enables animals to select and control interactions with their environment to achieve their goals and avoid negative outcomes. The phylogenetic perspective suggests that the primate posterior parietal cortex is part of a topographically organized dorsomedial neocortical system for visually guided interactions oriented with respect to objects in the world (Cisek, 2007). This system is organized as parallel sensorimotor streams, each contributing to a specific type of action within the animal’s behavioral repertoire, whose activity is orchestrated through selective invigoration, energization, or drive from the basal ganglia and other structures (Cisek & Thura, 2018; Grillner, Robertson, & Stephenson-Jones, 2013). Within each of these fronto-parietal action streams, target selection occurs through winner-take-all dynamics taking place in an idiosyncratic spatial reference frame specific to each given type of action (e.g., retinotopic for eye movements). One of those streams is concerned with orienting gaze through eye and head movements, and appears to have an executive role simply because so many of the other streams rely on high-resolution visual information that is derived from the fovea. When an animal (including humans) is placed in a laboratory situation and trained/instructed to perform just one isolated aspect of complex natural behavior, what the researcher will observe in this region is activity that appears to be related to what the researcher has defined as “attention,” “intention,” or “decision-making,” depending on the particular task variables that are being experimentally manipulated. But it does not follow from these correlations that there exists anything in the brain that can be meaningfully delineated as an “attentional system” (or, for that matter, an “intention system” or “decision system”). The key insight is that the posterior parietal cortex is not part of an “attention system” but, rather, that selective attention phenomena are part of what the posterior parietal cortex produces as it goes about its business of controlling goal-directed action.

## Summary and conclusions

We thus conclude that selectivity emerged through evolution as a design feature to enable efficient goal-directed action. Such selectivity became necessary as the action repertoire of the given line of organisms that led to humans increased. This means that selectivity is an emerging property arising from a myriad underlying processes, and the simple fact that humans (and other species showing selective attention) evolved the way they did, with selective attention being one of many byproducts, next to “selective intention” and “selective decision making.” Here, we have primarily emphasized selection mechanisms in the superior colliculus and parietal cortex, but similar arguments can be made for other selection mechanisms in other brain regions. For example, Krauzlis et al. (2014) suggest how some types of “attentional” phenomena could be products of value-based selection mechanisms of the basal ganglia. If selectivity is a design feature that emerged as the repertoire of behaviors increased in number and sophistication (avoidance, approach, saccade, eat, reach, grasp, use tools…), it would seem futile to search for a single dedicated functional or neural subsystem generating selection. We feel that this futility is the reason that attention research has so many longstanding and rather fruitless debates about the true origins and processes of selective attention. These debates are commonly binary in nature because the debates start with the assumption of one cause or singular central core system. As this one cause is then increasingly challenged by additional research findings, another, commonly opposite cause is established… and the process and debate continues. We suspect that none of these debates will come to an end, simply because the proponents of all camps are “correct” in some way and in some cases given that selectivity is a feature of the system that has emerged from the interaction of many factors across evolution.

And yet, we strongly feel that these debates do not move our field forward; that they do not really increase our understanding of how “attentional phenomena” are generated. Pursuing the analytic approach and trying to use one concept like “attention” to explain all of these results (that is, as a singular explanans) is problematic – the term invariably gets spread so thin, across so many different findings, that it ends up being too vague to have any empirical punch. Researchers are right to pursue these as multiple explananda, but would be wrong to seek or be forced to rule out only one explanans. Rather, inasmuch as it is possible, one should seek to identify the key mechanisms and processes at work and explain each in turn.

In an analytic approach to science, one runs the risk of becoming a slave to the concepts that have been generated. Many researchers have taken terms like “attention,” “intention,” and “decision making” from everyday language and expect this linguistic categorization to somehow map to identifiable mechanisms in the brain or functions. Of course, when one starts to peer into actual neural functions, there is no clear delineation, only a set of processes that interact to create selectivity in the end. These processes interact not because they belong to a dedicated system, but because the human brain and body evolved this way and selectivity was a necessary feature to achieve efficient behavior. Further, everything an individual does throughout their life (distant and recent past) creates, reinforces, and shapes selection: Turning to the left makes us ignore stimuli on the right, picking one apple makes us overlook the others, saying one word prevents us from uttering any other. And each of the different selections results in all ranges of rewards, from positive gains to negative losses. Selection and reward are thus inherent ingredients of all our lives and the way we lead them (Allport, 1987).

To produce selective behavior, multiple, inter-related processes integrate numerous sources of information. One of the challenges is that these processes unfold over different timeframes (e.g., Chapman et al., 2015b; Welsh, Neyedli, & Tremblay, 2013). Therefore, in a laboratory setting, if these processes are only observed during one point or snapshot during the selection process, the observation could appear to reflect “attention” or “intention” or “decision making and reward.” The synthetic approach proposed here also rectifies and makes explicit that reward and selection history are intertwined subjects, but likely reflect multiple processes that contribute to goal-oriented behavior. For example, the synthetic approach can account for harm avoidance. Specifically, harmful stimuli should receive priority processing for detection, yet the organism should move away from these stimuli. The primitive neural circuits for reward/approach and harm/avoid processes diverge early in evolutionary history, providing a process-based account for divergent findings regarding positive and negative value-based stimuli. Likewise, the synthetic approach explains why sensitivity to different features of objects depends on the action context (Bekkering & Neggers, 2002; Craighero et al., 1999; Fagioli et al., 2007; Welsh & Pratt, 2008) – because the context determines which action-centered parietal stream, with its idiosyncratic representation of the external world, is being selectively invigorated at a given time.

One of the great conundrums in experimental psychology and neuroscience is exactly how all of these streams of information diverge from initial sensory areas and then converge to produce action. Working backwards from what researchers observe in behavior, it is known that generally only one goal-directed movement is performed at a time, though more than one might be simultaneously represented (e.g., Cisek & Kalaska, 2005). As discussed in the section on evolutionary adaptations, we advocate for a parallel competitive structure with winner-take-all dynamics resolving to produce a single action for each action system (e.g., hand and eye). Of course, much of the detail about how this occurs is an open question and beyond the scope of this article. What we hope to emphasize here is the synthetic approach to understanding how complex sensory information is transformed into action. The corollary argument is that progress is hindered when we appeal to or attempt to apply catch-all terms like “attention.” Thus, rather than saying that an individual “pays more attention to a physically salient stimulus,” one should make an attempt to understand the mechanism by which physical salience translates to more efficient processing and behavior. Instead of arguing that rewarding stimuli “demand more attention,” provide a description of how a particular reward is associated with a particular target, and how, perhaps even more astoundingly, the cognitive system/brain then recalls this association in a fraction of a second to guide behavior on a subsequent trial. Experiment to figure out how and why visual information presented at a location selected for action is amplified, rather than passing the finding off as “just attention.” Hence, turn to the mechanisms that we understand and try to re-create the behavior that cognitive and neural scientists are interested in. If that approach turns out to be successful, there will be no need for undefinable concepts like attention, either in describing the explanandum or in describing the explanans.

The synthetic approach we suggest here might appear reductionist. On the one hand, we emphasize that the approach we propose does not favor neural over functional explanations. True cognitive neuroscience relies on the idea that good theories should take both neural and functional constraints into account, so that neural and functional theories do not contradict each other. This does not imply, and actually logically undermines, the sometimes observed tendency to consider neural explanations as somehow more fundamental or causal than functional explanations. Even though many of our examples referred to neural findings and accounts, and even though our evolutionary reasoning was couched mainly in neural terms, we do not advocate any primacy of neural over functional explanations, and have strived to provide evidence from both approaches. On the other hand, however, we fully subscribe to the assumption that good theories in cognitive psychology and the cognitive neurosciences come from testable hypotheses of how an observed phenomenon (the explanandum) is produced by its underlying mechanisms (the explanans), irrespective of whether these mechanisms are described in neural or functional terms. In contrast to mainstream research, our synthetic approach requires the theorist to reconstruct a phenomenon from well-understood basic mechanisms, rather than analyzing the phenomenon into pieces. Our expectation is that this synthetic/constructivist approach will eventually reveal that our original ways to delineate the phenomena we aim to explain were misleading, and we feel that this is in particular true for the concept of attention. Hence, we argue that, in contrast to James’s (1890) assertion, no one knows, or can ever know, exactly what attention is.

## Footnotes

1 Note that William James’ own approach to attention was mainly concerned with phenomenology, an aspect of attention that we do not further consider in this article given the modern functional and neural approaches we address instead.
